# Molecular docking analysis of imeglimin and its derivatives with estrogen receptor-alpha

**DOI:** 10.6026/973206300200711

**Published:** 2024-07-31

**Authors:** Anitha Elango, Iyanar Kannan, Ramya Ravichandar, Punnagai Kumaravelu

**Affiliations:** 1 Department of Pharmacology, Panimalar Medical College Hospital and Research Institute, Chennai; 2 Department of Microbiology, Tagore Medical College and Hospital, Chennai; 3 Department of Pharmacology, Tagore Medical College and Hospital, Chennai

**Keywords:** Imeglimin, anticancer agents, molecular docking, *in silico*, drug discovery, computational methods

## Abstract

Estrogen receptor-α (ER- α) is a principal endocrine regulatory protein in breast cancer. The progression of ER-α positive breast cancer is slowed by selective estrogen receptor modulators such as Tamoxifen. But, long term therapy with Tamoxifen leads to resistance. Therefore, it is of interest to document the Molecular docking and pharmacokinetic analysis of imeglimin derivatives with ER-alpha. Among the 166 derivatives of Imeglimin, only five derivatives were shortlisted after toxicity testing. The selected derivatives showed good binding affinity with favorable pharmacokinetic profiles. The selected compounds of Imeglimin were found to possess excellent anticancer potential and could be considered as novel, cost-effective anticancer agents effective against ER positive breast cancer for further investigation.

## Background:

Breast cancer is a significant worldwide health concern. One in every 8 females is predicted to be diagnosed with invasive breast cancer in their lifetime [2]. Breast cancer has been treated with a variety of chemotherapeutic drugs, hormonal agents, and targeted drugs. The patient is severely incapacitated by the adverse effect profile of the various drugs classes [3]. Furthermore, a significant proportion of the population from lower socioeconomic groups has poor compliance with therapy due to its high cost. The growth and spread of breast cancer, especially hormone receptor-positive breast cancer, are significantly influenced by estrogen receptors (ERs) [4]. Apart from the effects on cancer, binding of chemical substances to the estrogen receptor (ER) can lead on to endocrine disruption [5]. ER-alpha (ERα) and ER-beta (ERβ) are the two primary forms of estrogen receptors. ERα is the most prevalent form found in breast tissue and is more frequently linked to breast cancer [6]. Estrogen receptor-α (ERα) is a 66 kDa protein having a ligand binding domain of 245 residues [7]. ER-α is one of the 48 nuclear intracellular receptors [8]. It is one of the two major receptors for the endogenous estrogen, 17β-estradiol (E2) [7]. ERs are also present in the plasma membrane and mitochondria [9]. The transcriptional activities of ER- α can be stimulated by second messenger pathways [10]. The altered expression of ERα can be detected in breast cancer using aptamers [11]. The progression of ER-α positive breast cancer is slowed by selective estrogen receptor modulators such as Tamoxifen. But, long term therapy with Tamoxifen leads to resistance [1]. Moreover, Tamoxifen leads to increased risk of endometrial cancer, stroke and pulmonary embolism owing to its estrogen agonistic action at certain organs [12]. Hence, finding new and better drugs is crucial because it is still challenging to address such problems. Recently, Imeglimin, a promising medication for diabetes, structurally similar to Metformin, has drawn attention for its complex pharmacological profile, which includes anti-inflammatory and anti-proliferative properties [13]. The investigation of Imeglimin and its derivatives in breast cancer is a paradigm-shifting step in pharmaceutical research that could lead to the introduction of a new class of medications for the treatment of this common malignancy. Therefore, it is of interest to document the Molecular docking and pharmacokinetic analysis of Imeglimin derivatives with ER-alpha.

## Materials and methods:

## Ligand preparation:

The structure of the ligand (Imeglimin) was obtained from PUBCHEM database (PubChem CID: 24812808). The chemical structures of various derivatives of Imeglimin were drawn using Chemsketch software. The designed structures were then optimised using Openbabel software. A total of 166 derivatives were designed and the bond lengths and angles were standardized using the clean structure command. In addition, IUPAC name was added using the software [14].

## Toxicity Prediction and Pharmacokinetic analysis:

Once the derivatives were developed, simplified molecular input line entry system (SMILES) were created using swissADME, an online tool from Swiss Institute of Bioinformatics (SIB). (http://www.swissadme.ch/) [15]. They were screened for various toxicity parameters such as AMES toxicity, Acute oral Toxicity, Carcinogenicity and Rat acute toxicity LD50 using pkCSM. (http://biosig.unimelb.edu.au/pkcsm/) [16]. Only those derivatives which exhibited least toxicity were included for further research. Using the pharmacokinetic parameters, the compounds were then scanned for "Lipinski's rule of 5". The shortlisted compounds that did not violate "Lipinski's rule of 5" were then chosen for further study and analysis [17].

## Target selection:

The target protein structure was retrieved using RCSB protein databank. Estrogen receptor-alpha (ER-α) (PDB ID: 1A52) was chosen as our receptor target.

## Molecular docking:

**Figure 1:**
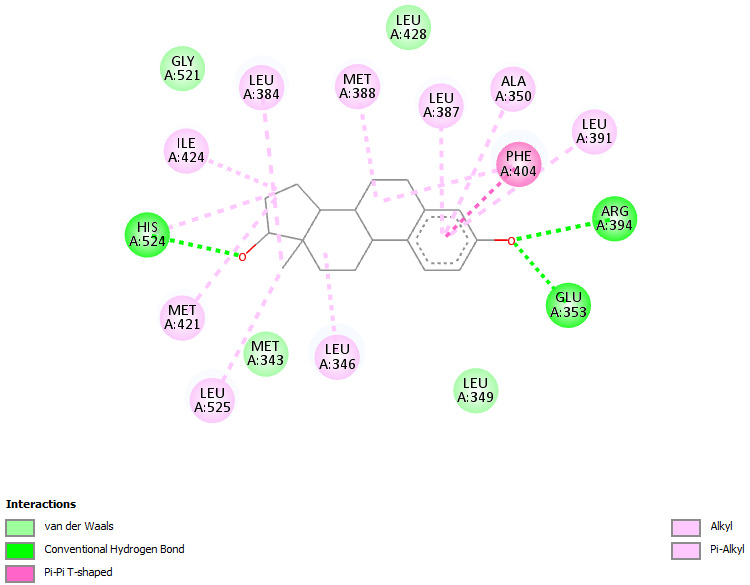
Amino acids of the protein target Estrogen receptor-alpha (ER-α) showing significant bonds and interactions with Tamoxifen generated using Discovery studio software.

Protein- ligand interaction analysis is a vital step in virtual screening of potential ligands [18]. Discovery studio software was used for initial docking analysis. Receptor ligand interactions were checked and the amino acids that showed significant interaction using hydrogen bond, vanderwaals, etc. were noted (Figure 1). The water molecules and ligands bound to the target molecule (ER- α) were removed and a clean structure was saved as a PDB file.

**Figure 2:**
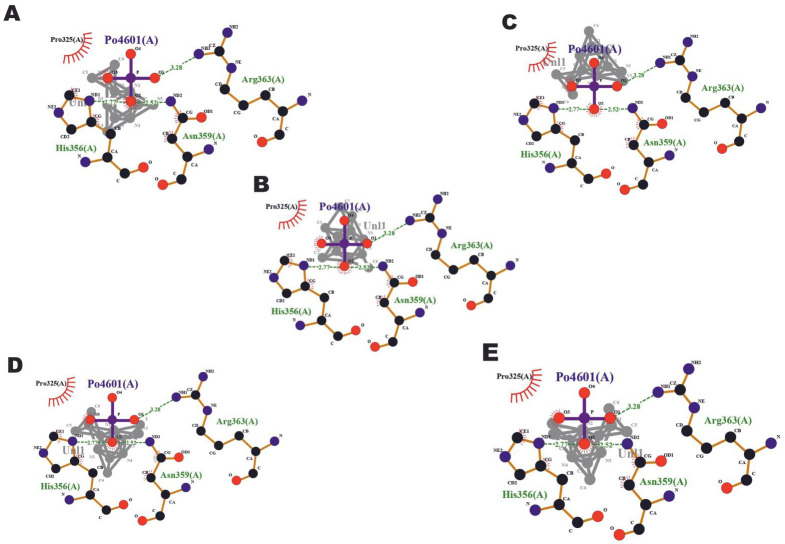
Final docking pose of the molecules with protein target Estrogen receptor-alpha (ER-α) generated using Ligplot software: A) (4S)-6-N, 6-N-dichloro-2-N,2-N,4-trimethyl-1,4-dihydro-1,3,5-triazine-2,6-diamine; B) (2R)-N,2,5,6-tetramethyl-2H-1,3,5-triazin-4-amine; C) (4S)-6-N-chloro-2-N,2-N,4-trimethyl-1,4-dihydro-1,3,5-triazine-2,6-diamine; D) (4S)-6-N-fluoro-2-N,2-N,4-trimethyl-1,4-dihydro-1,3,5-triazine-2,6-diamine; E) (4S)-6-hydrazinyl-N,N,4-trimethyl-1,4-dihydro-1,3,5-triazin-2-amine

PyRx software was then used for auto docking. Both the ligand and target molecule were converted into pdbqt format. Using autodock wizard, docking of ligand with the target was performed and the output was obtained. Among the ligand derivatives, the ones with RMSD <3 Å and higher binding affinity were selected. According to the study done by Gonzalez TL *et al*, RMSDs of ligands docked into human, mouse and rat ER-α were 0.49 Å (human-mouse), 1.19 Å (human-rat) and 0.18 Å (mouse-rat) [19]. Using Ligplot+ software, the final docking pose of each derivative with the target protein was obtained (Figure 2).

## Results and Discussion:

**Figure 3:**
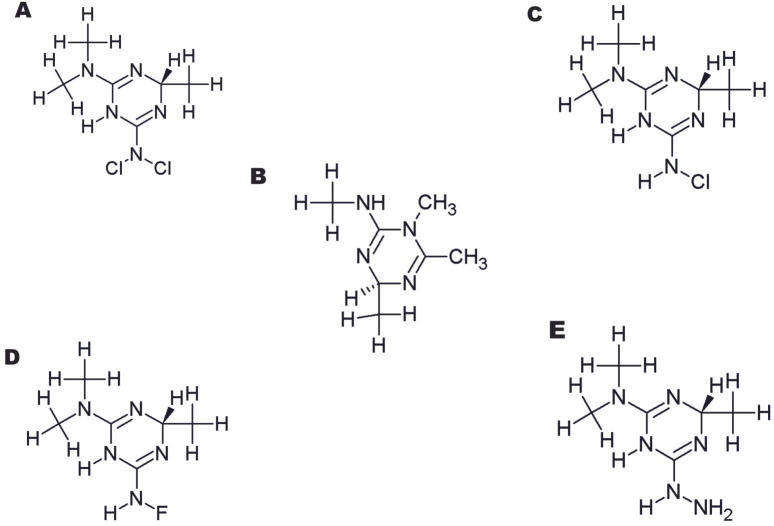
2D structures of the finalized structures of Imeglimin and its derivatives generated using Chemsketch software. A) (4S)-6-N,6-N-dichloro-2-N,2-N,4-trimethyl-1,4-dihydro-1,3,5-triazine-2,6-diamine; B) (2R)-N,2,5,6-tetramethyl-2H-1,3,5-triazin-4-amine; C) (4S)-6-N-chloro-2-N,2-N,4-trimethyl-1,4-dihydro-1,3,5-triazine-2,6-diamine; D) (4S)-6-N-fluoro-2-N,2-N,4-trimethyl-1,4-dihydro-1,3,5-triazine-2,6-diamine; E) (4S)-6-hydrazinyl-N,N,4-trimethyl-1,4-dihydro-1,3,5-triazin-2-amine

A total of 166 derivatives of Imeglimin were developed. After toxicity testing, only five derivatives were shortlisted after excluding the toxic derivatives. Figure 3 depicts the 2D structures of the finalized structures of Imeglimin and its derivatives. All the five derivatives of Imeglimin with the Estrogen receptor-alpha (ER-α) showed the binding energies ranging between -6.4 to -7 Kcal/mol which were comparable with that of Tamoxifen (-6.68 Kcal/mol) (Table 1). Study by Ahmed et al showed good binding affinity of quinazolinone compounds to ER- α with maximum inhibition of 85% comparable to Tamoxifen (100%) [20]. It is interesting that dihydrotestosterone (DHT), being an endogenous androgen hormone, has the ability to bind to the ER- α with a relative binding affinity of 0.03% as compared to E2 [21]. All the compounds showed three hydrogen bond interactions each with Asn359, His356 and Arg363 of ER-α with a distance of 2.52, 2.77 and 3.28 Å respectively. Elshal *et al.* showed that the amino acids Leu A308, Thr C334, Val A368, Thr A371 in the ERα protein interacted with a lectin protein, Concanavalin-Aa. There was a good interaction between Concanavalin-Aa and ERα proving its antagonistic effect on ERα and also showed synergistic action with Tamoxifen in breast cancer [22]. In the study by Masand *et al.*, estrogen receptor alpha binders were analyzed for hormone dependent forms of breast cancer. Among the compounds analyzed by molecular docking, CHEMBL304552 was found to exhibit hydrogen bonds with Glu419 and Arg394 with a distance of 3.55 and 3.03 Å respectively [23]. In the study by Lu Q *et al*, the binding between HO-PBDEs and ERα were van der Waals and electrostatic interactions and also the hydrogen bonds between the residues Glu353, Gly521 and ligands were essential for securing the ligands into the active site of ERα and stabilization of their conformations [24]. The hydrogen bond helps to assess the inhibitor's efficacy against the target protein and maintains the stability of the complex [25]. A study done by Patidar *et al.* tested 40 inhibitors of mTOR receptor protein against breast cancer, among which SF1126 showed the best docking score of -8.705 [26]. According to Marwa F Ahmed *et al.*, a series of quinazoline derivatives were synthesized and docking analysis was done against estrogen receptor alpha. All the docked compounds showed binding energy ranging between -13.5 and -25.3 kcal/mol [27].

After 166 Imeglimin derivatives were synthesized and subjected to toxicity screening, five non-toxic candidates were selected and potentially toxic derivatives were eliminated. Assessment of ADMET properties by experimental evaluation requires expense and time. Computational approach to analyze pharmacokinetic (ADME) and toxicity properties leads to the prompt and cost-effective generation of drug candidates [16]. Accordingly, pharmacokinetic assessment of these derivatives was done and a bioavailability score of 0.55 was obtained. This suggests that the compounds have strong systemic absorption and favorable pharmacokinetic properties. Furthermore, Lipinski's rule of five was applied to evaluate their drug-likeness, with no violations observed. Other rules of drug likeness related to physicochemical/ pharmacokinetic parameters such as Ghose, Veber, Egan, and Muegge rules were also applied and the candidates showed satisfactory drug-likeness properties (Table 2). According to the study done by Warude *et al.*, indole based benzamides showed only one violation as per Lipinski's rule of 5 and a bioavailability score of 0.55 was obtained [28].

Acute toxicity studies were performed to analyse the toxicological profile of Imeglimin and its derivatives. The Ames test is a renowned in vitro bacterial mutagenicity assay used to determine the genotoxic potential of substances [29]. The negative results obtained from the Ames test for Imeglimin and its derivatives indicate that they possess no mutagenic activity. This implies a minimal risk of inducing mutations in bacterial DNA under the tested conditions. This reinforces the idea that Imeglimin is not genotoxic, which is an important consideration when assessing the safety of intended for therapeutic use. Based on the results of the acute oral toxicity, Imeglimin and its derivatives have an estimated fatal dose for 50% of the tested population (LD50) ranging from 0.4043 to 0.5416. This relatively low LD50 value supports the placement of the compounds into Toxicity Class III. Substances of the toxicity class III are defined as having moderate toxicity, with LD50 values usually falling between 0.1 and 1.0 g/kg body weight. Imeglimin and its derivatives fall into this range, suggesting a moderate degree of acute toxicity. Furthermore, it is reassuring to find out that preclinical research has not shown any carcinogenic characteristics. Carcinogenicity studies provide critical insights into the potential long-term risks associated with exposure to a substance. The lack of carcinogenic effects observed indicates that there is no increased risk of cancer development, further supporting their safety profile (Table 3).

The efficacy and safety of imeglimin derivatives as possible therapeutic agents are largely dependent on their pharmacokinetic properties. The data obtained about their pharmacological characteristics is significant and includes information on their absorption, permeability, plasma protein binding, and inhibition of cytochrome P450 (CYP) enzymes, skin penetration and clearance. Imeglimin derivatives showed high gastrointestinal (GI) absorption which suggests that these compounds are efficiently absorbed from the GI tract into the systemic circulation. This is a favorable characteristic for oral medications, as it indicates a high bioavailability and potential for effective therapeutic action. The absence of blood-brain barrier (BBB) penetration is an important finding because it implies that derivatives of Imeglimin are unlikely to cross the BBB to have an impact on the central nervous system (CNS). This characteristic may lower the possibility of CNS-related side effects, which could improve their safety profile. Plasma protein binding can impact the distribution, metabolism, and elimination of drugs in the body. The observed range of plasma protein binding for Imeglimin derivatives is 7.571% to 22.203%. This is below the optimum plasma protein binding limit of 90%. High plasma protein binding correlates with low therapeutic index. The lack of inhibition of CYP 1A2, CYP 2C19, CYP 2C9, CYP 2D6 and CYP 3A4 by Imeglimin derivatives is a positive finding, as it indicates that these compounds are unlikely to interfere with the metabolism of other drugs that are substrates for these CYP enzymes. This suggests a low potential for drug-drug interactions involving these pathways. Imeglimin derivatives were found to exhibit a range of skin permeation (-7.23 to -8.28), indicating variable degrees of permeability through the skin. Skin permeation is relevant for topical formulations and can influence the effectiveness of transdermal delivery systems. The range of clearance observed for Imeglimin derivatives (6.015 to 7.513 ml/min/kg) indicates moderate range of clearance from the body. Clearance is an important pharmacokinetic parameter that influences the dosing regimen and overall exposure of an individual to a drug. In summary, the pharmacokinetic parameters of Imeglimin derivatives, including their absorption, permeation, plasma protein binding, inhibition of CYP enzymes, skin permeation, and clearance, provide significant insights into their potential as therapeutic agents. These findings suggest that Imeglimin derivatives have favorable pharmacokinetic profiles, with efficient GI absorption, minimal BBB permeation, low plasma protein binding, no inhibition of major CYP enzymes, and variable skin permeation and clearance rates (Table 4).

Breast cancer is one of the most prevalent types of malignancies among females. The activation of ERα by oestrogens is commonly attributed to increased proliferation in many breast malignancies [30]. 70% of breast cancers are ER-α positive and many patients showed intrinsic resistance to hormonal treatment [31]. Potential non-hormonal therapeutic agents that alter ER-α are now being investigated for the management of breast cancer. Metformin is a member of the biguanides group and is useful in treating type 2 diabetes mellitus as well as many cancers, including breast cancer, according to several studies [32]. Metformin reduces insulin levels and modifies the AMPK/mTOR/P70S6K pathway to produce anticancer effects [33]. EGFR downregulation, p53 phosphorylation, cell cycle arrest, and induction of apoptosis are caused by AMPK activation [34]. Scordamaglia *et al.* showed that metformin inhibits the activation of transduction pathways and proliferative changes in breast cancer cells mediated by the insulin receptor [35].

A newly developed drug called Imeglimin shares structural similarities with metformin. In Japan, Imeglimin was initially approved for the treatment of type 2 diabetes mellitus in 2021. Additionally, Imeglimin demonstrates AMPK activation, which is accountable for its antiproliferative action. According to Hozumi *et al.*, there is no significant difference between the effects of Metformin and Imeglimin on AMPK phosphorylation [36]. While some research has been done using hepatocellular carcinoma cells, there are no prior studies that have been published in the literature to support Imeglimin's anticancer properties with respect to breast cancer. Hence this molecular docking study was undertaken to ascertain the anticancer effects of Imeglimin and its derivatives in breast cancer. After testing for toxicity, five non-toxic candidates were selected among 166 derivatives of Imeglimin, thus excluding the toxic derivatives. Molecular docking showed that all the five derivatives of Imeglimin formed three hydrogen bonds with the important residues of the estrogen receptor-alpha (ER-α). They also exhibited favorable binding energies with ER-α, comparable to that of tamoxifen. The selected derivatives showed a bioavailability score of 0.55, thus exhibiting excellent pharmacokinetic characteristics and substantial systemic absorption. Observation of no violations of Lipinski's rule of five suggests good drug-likeness.

Toxicity studies such as AMES test, acute oral toxicity (LD50) and carcinogenicity were performed. The Ames test revealed no mutagenicity. Based on the LD50 values, Imeglimin and its derivatives were classified into toxicity Class III (moderate toxicity). Preclinical studies proved the absence of any carcinogenic characteristics. Hence these studies provide valuable inputs regarding the safety profile of Imeglimin and its derivatives. Analysis of various pharmacokinetic characteristics showed good gastrointestinal absorption, indicating a high degree of bioavailability, no permeation of blood-brain barrier (BBB), limited plasma protein binding, no inhibition of major CYP enzymes (1A2, 2C19, 2C9, 2D6, 3A4) and moderate range of clearance. In silico techniques are very crucial to establish the 3 R concept- Reduction, Replacement and Refinement. This is useful as an alternative to animal experiments and also reduces the large expenditure involved in research [37]. Molecular docking has been a successful approach for determining realistic inhibition mechanisms and ligand- protein interactions. More negative the binding energy (BE), more effective is the ligand binding to the target protein. Spiriti et al have developed a flexible docking approach based on mixed-resolution Monte Carlo (MRMC), to offer a balance with speed, protein flexibility and sampling power [38]. Using molecular docking, the selected Imeglimin derivatives exhibited substantial binding (lower BE) with the ER-α comparable with Tamoxifen. This denotes that these compounds may find application as ER-α inhibitors against breast cancer.

## Conclusion:

Five non-toxic derivatives of Imeglimin with favorable pharmacokinetic profiles are identified for further consideration as anticancer agents.

## Figures and Tables

**Table 1:** Protein ligand interactions between the selected ligands

**Selected ligands**	**Binding energy kCal/mol**	**Interaction residues**	**Hydrogen bonds**	**Hydrogen bond distance in Å**
(4S)-6-N,6-N-dichloro-2-N,2-N,4-trimethyl-1,4-dihydro-1,3,5-triazine-2,6-diamine	-6.4	His356, Pro325, Arg363, Asn359	N-O Asn359 N-O His356 N-O Arg363	2.52 2.77 3.28
(2R)-N,2,5,6-tetramethyl-2H-1,3,5-triazin-4-amine	-6.5	His356, Pro325, Arg363, Asn359	N-O Asn359 N-O His356 N-O Arg363	2.52 2.77 3.28
(4S)-6-N-chloro-2-N,2-N,4-trimethyl-1,4-dihydro-1,3,5-triazine-2,6-diamine	-6.9	His356, Pro325, Arg363, Asn359	N-O Asn359 N-O His356 N-O Arg363	2.52 2.77 3.28
(4S)-6-N-fluoro-2-N,2-N,4-trimethyl-1,4-dihydro-1,3,5-triazine-2,6-diamine	-7	His356, Pro325, Arg363, Asn359	N-O Asn359 N-O His356 N-O Arg363	2.52 2.77 3.28
(4S)-6-hydrazinyl-N,N,4-trimethyl-1,4-dihydro-1,3,5-triazin-2-amine	-6.6	His356, Pro325, Arg363, Asn359	N-O Asn359 N-O His356 N-O Arg363	2.52 2.77 3.28

**Table 2:** Drug likeness of the selected ligands

	**No. of violations**					
**Ligand**	**Lipinski**	**Ghose**	**Veber**	**Egan**	**Muegge**	**Bioavailability score**
(4S)-6-N,6-N-dichloro-2-N,2-N,4-trimethyl-1,4-dihydro-1,3,5-triazine-2,6-diamine	0	0	0	0	0	0.55
(2R)-N,2,5,6-tetramethyl-2H-1,3,5-triazin-4-amine	0	2	0	0	1	0.55
(4S)-6-N-chloro-2-N,2-N,4-trimethyl-1,4-dihydro-1,3,5-triazine-2,6-diamine	0	1	0	0	1	0.55
(4S)-6-N-fluoro-2-N,2-N,4-trimethyl-1,4-dihydro-1,3,5-triazine-2,6-diamine	0	1	0	0	1	0.55
(4S)-6-hydrazinyl-N,N,4-trimethyl-1,4-dihydro-1,3,5-triazin-2-amine	0	1	0	0	1	0.55

**Table 3:** Estimated values of acute toxicity for the selected ligands

	**AMES toxicity**	**Acute oral Toxicity class**	**Carcinogenicity (Three class)**	**Rat acute toxicity LD50 (mol/kg)**
		**III**	**Non carcinogenic**	
(4S)-6-N,6-N-dichloro-2-N,2-N,4-trimethyl-1,4-dihydro-1,3,5-triazine-2,6-diamine	No	0.4043	0.4661	2.8347
(2R)-N,2,5,6-tetramethyl-2H-1,3,5-triazin-4-amine	No	0.4811	0.4701	2.5432
(4S)-6-N-chloro-2-N,2-N,4-trimethyl-1,4-dihydro-1,3,5-triazine-2,6-diamine	No	0.487	0.4844	2.7168
(4S)-6-N-fluoro-2-N,2-N,4-trimethyl-1,4-dihydro-1,3,5-triazine-2,6-diamine	No	0.5003	0.5045	2.732
(4S)-6-hydrazinyl-N,N,4-trimethyl-1,4-dihydro-1,3,5-triazin-2-amine	No	0.5416	0.4415	2.6048

**Table 4:** Pharmacokinetic properties of the selected ligands

Parameters	(4S)-6-N,6-N-dichloro-2-N,2-N,4-trimethyl-1,4-dihydro-1,3,5-triazine-2,6-diamine	(2R)-N,2,5,6-tetramethyl-2H-1,3,5-triazin-4-amine	(4S)-6-N-chloro-2-N,2-N,4-trimethyl-1,4-dihydro-1,3,5-triazine-2,6-diamine	(4S)-6-N-fluoro-2-N,2-N,4-trimethyl-1,4-dihydro-1,3,5-triazine-2,6-diamine	(4S)-6-hydrazinyl-N,N,4-trimethyl-1,4-dihydro-1,3,5-triazin-2-amine
GI absorption	High	High	High	High	Low
BBB permeation	No	No	No	No	No
P-gp substrate	No	No	No	No	No
Plasma protein binding	22.20%	7.57%	9.23%	7.57%	8.15%
CYP 1A2 inhibition	No	No	No	No	No
CYP 2C19 inhibition	No	No	No	No	No
CYP 2C9 inhibition	No	No	No	No	No
CYP 2D6 inhibition	No	No	No	No	No
CYP 3A4 inhibition	No	No	No	No	No
Log Kp (skin permeation)	-7.23	-7.53	-7.58	-7.66	-8.28
Clearance	6.015	7.513	7.265	7.513	6.101
